# The Transformation of the Centrosome into the Basal Body: Similarities and Dissimilarities between Somatic and Male Germ Cells and Their Relevance for Male Fertility

**DOI:** 10.3390/cells10092266

**Published:** 2021-08-31

**Authors:** Constanza Tapia Contreras, Sigrid Hoyer-Fender

**Affiliations:** Göttingen Center of Molecular Biosciences, Johann-Friedrich-Blumenbach Institute for Zoology and Anthropology-Developmental Biology, Faculty of Biology and Psychology, Georg-August University of Göttingen, 37077 Göttingen, Germany; constanza.tapia-contreras@biologie.uni-goettingen.de

**Keywords:** centrosome, centriole, male fertility, acephalic spermatozoa syndrome, connecting piece, HTCA, sperm decapitation, capitulum, segmented columns, proteome

## Abstract

The sperm flagellum is essential for the transport of the genetic material toward the oocyte and thus the transmission of the genetic information to the next generation. During the haploid phase of spermatogenesis, i.e., spermiogenesis, a morphological and molecular restructuring of the male germ cell, the round spermatid, takes place that includes the silencing and compaction of the nucleus, the formation of the acrosomal vesicle from the Golgi apparatus, the formation of the sperm tail, and, finally, the shedding of excessive cytoplasm. Sperm tail formation starts in the round spermatid stage when the pair of centrioles moves toward the posterior pole of the nucleus. The sperm tail, eventually, becomes located opposed to the acrosomal vesicle, which develops at the anterior pole of the nucleus. The centriole pair tightly attaches to the nucleus, forming a nuclear membrane indentation. An articular structure is formed around the centriole pair known as the connecting piece, situated in the neck region and linking the sperm head to the tail, also named the head-to-tail coupling apparatus or, in short, HTCA. Finally, the sperm tail grows out from the distal centriole that is now transformed into the basal body of the flagellum. However, a centriole pair is found in nearly all cells of the body. In somatic cells, it accumulates a large mass of proteins, the pericentriolar material (PCM), that together constitute the centrosome, which is the main microtubule-organizing center of the cell, essential not only for the structuring of the cytoskeleton and the overall cellular organization but also for mitotic spindle formation and chromosome segregation. However, in post-mitotic (G1 or G0) cells, the centrosome is transformed into the basal body. In this case, one of the centrioles, which is always the oldest or mother centriole, grows the axoneme of a cilium. Most cells of the body carry a single cilium known as the primary cilium that serves as an antenna sensing the cell’s environment. Besides, specialized cells develop multiple motile cilia differing in substructure from the immotile primary cilia that are essential in moving fluids or cargos over the cellular surface. Impairment of cilia formation causes numerous severe syndromes that are collectively subsumed as ciliopathies. This comparative overview serves to illustrate the molecular mechanisms of basal body formation, their similarities, and dissimilarities, in somatic versus male germ cells, by discussing the involved proteins/genes and their expression, localization, and function. The review, thus, aimed to provide a deeper knowledge of the molecular players that is essential for the expansion of clinical diagnostics and treatment of male fertility disorders.

## 1. Introduction

The germinal epithelium of the testis ensures ongoing delivery of fertilization-competent spermatozoa. Continuous production of spermatozoa during spermatogenesis is based on three successive phases: proliferation, meiosis, and cytodifferentiation [1,2,3]. The proliferative phase corresponds first to a series of asymmetric cell divisions that ensures the constant supply of germ cells not only by maintaining the stem cell pool but also by generating spermatogonia that then further proliferate and, eventually, are transformed into the final type B spermatogonia undergoing a last cell division to enter meiosis [4]. Primary spermatocytes then undergo the first meiotic division characterized by chromosome condensation, synapsis, and recombination, and finally separation of homologous chromosomes generating diploid secondary spermatocytes. The second meiotic division then follows immediately, ensuring DNA reduction and the generation of haploid germ cells, the spermatids. Thus, due to the two meiotic divisions, four haploid spermatids are generated out of each spermatocyte that has entered meiosis. Haploid spermatids that are the direct product of the second meiotic division are spherical and not resembling mature spermatozoa. In the final cytodifferentiation phase, in spermiogenesis, round spermatids undergo a dramatic remodeling that includes nuclear condensation and reshaping, acrosome and flagellum development, and shedding of excessive cytoplasm, thus generating the mature and fertilization-competent spermatozoon of well-known morphology [2,3,5,6].

Spermatozoa exclusively function in delivering the genetic information to the oocyte, initiating and supporting the development of a new generation. The differentiation process that gives rise to the mature spermatozoon thus serves to generate a streamlined and motile cell able to reach and fertilize the oocyte. Spermiogenesis is subdivided into the four successive phases referring to the development of the acrosomal vesicle: (1) Golgi phase, (2) cap phase, (3) acrosome phase, and (4) the final maturation phase. The acrosome develops by the fusion of Golgi-derived vesicles that eventually attaches to the nuclear envelope forming a cap-like structure at the anterior part of the nucleus. Opposed to the acrosome, the flagellum is inserted into the nucleus at the posterior pole. Thus, the bipolarity of the spermatozoon is established by the position of the acrosome and the flagellum [7]. Formation of the sperm flagellum is initiated by the centriolar pair that is first located close to the Golgi apparatus but then migrates toward the caudal area. The centriolar pair initiates flagellum formation as well as the insertion of the flagellum into the nucleus, forming a tight linkage, the connecting piece or HTCA. The tight connection between sperm head and tail is crucial for the delivery of the sperm nucleus to the oocyte and successful fertilization, as revealed by infertile men suffering from sperm decapitation syndrome [8].

The assembly of the flagellum requires the availability of molecular components whose delivery is enabled by the intramanchette transport (IMT) and the intraflagellar transport (IFT) [9,10,11]. The manchette is a transient microtubule structure that develops during the acrosomal phase and disintegrates in the late stages of spermiogenesis [12,13]. The manchette is attached to the nucleus via the perinuclear ring, emanating its parallel-oriented microtubules toward the posterior pole, and forming a skirt-like structure surrounding the nucleus [14]. The manchette not only serves as a track for the delivery of flagellar components via IMT but also is essential for the reshaping of the spermatid nucleus to finally generate its species-specific sperm morphology.

The maturation phase is finalized by the completion of mature spermatozoa characterized by the flagellated sperm head. The sperm flagellum comprises the axoneme, the typical structure of eukaryotic cilia and flagella consisting of nine peripheral doublet microtubules and two centrally located singlet microtubules (9 × 2 + 2 arrangement), together with accessory structures. While the axoneme extends throughout the length of the sperm tail, the accessory outer dense fibers (ODFs) run in parallel to the axonemal doublets in the mid-piece of the sperm tail and throughout most of the principal piece. Axoneme and ODFs are, additionally, surrounded by the fibrous sheath (FS) in the principal piece and mitochondria in the mid-piece [7]. The ODFs most likely function in force transmission of the flagellar beat and in protecting the flagellum against shearing forces [15,16].

Malformation of spermatozoa, characterized by morphological and motility disorders in terato- and asthenozoospermia, respectively, causes male infertility. Although in vitro fertilization methods are often successful in overcoming male factor infertility, the prognostic significance is nevertheless variable for different sperm pathologies. Therefore, identification of the underlying genetic factors is essential not only for infertility diagnosis but also for fertility prognosis in assisted reproduction and for the ability to adequately inform patients of treatment outcomes and risks. Applying assisted reproduction techniques for the treatment of genetically induced male infertility bears the risk for the transmission and propagation of infertility to the offspring. Moreover, sperm head and neck anomalies seriously affect fertility prognosis [17]. The neck region is incorporated into the zygote during fertilization and provides the centrioles. Since in most animals, centrioles are eliminated during oogenesis, the sperm centrioles are essential for the formation of centrosomes and the early embryonic development [18]. Thus, abnormalities of the neck region are presumably causative for fertilization failure, abnormal cleavage of the embryo, and early pregnancy loss in humans [19,20,21].

The neck region of the spermatozoon is a highly modified derivative of the centriolar pair. To better understand the development of the HTCA and its clinical-relevant malformations, knowledge of its molecular composition is mandatory. As centrosomes are ubiquitous cellular organelles that are transformed into basal bodies when cilia formation starts, a closer look at their protein composition might be helpful to shed light on the HTCA and its components.

## 2. The Centrosome in Somatic Cells and Its Transformation into the Basal Body

**Figure 1:**
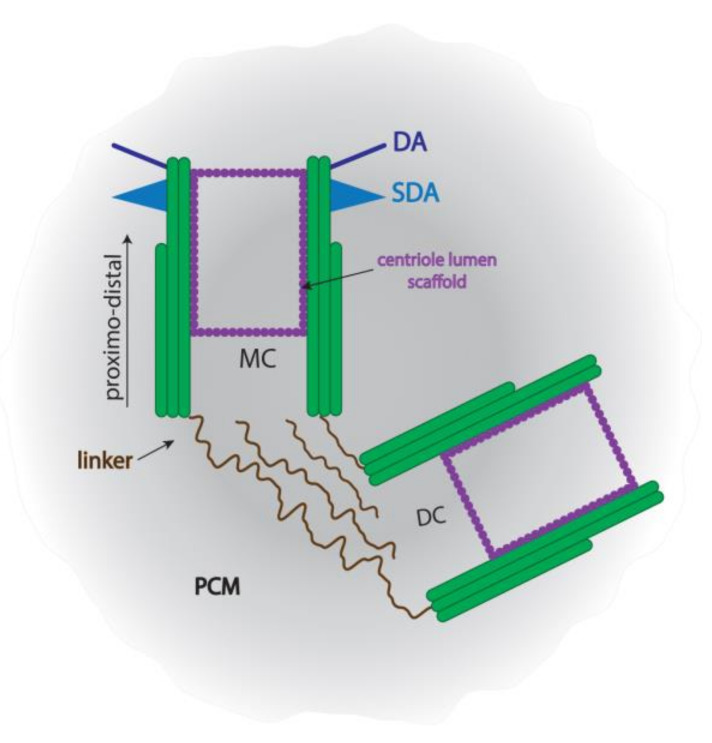
Depiction of a typical centrosome. The centriole pair is located in the center of the pericentriolar material (PCM). Mother and daughter centrioles, MC and DC, respectively, have a defined proximo-distal axis and are interconnected by linker proteins at their proximal ends. Distal and subdistal appendages, DA and SDA, respectively, extend from the distal end of the mother centriole. A centriole scaffold located at the inner wall of both centrioles stabilizes the microtubules.

A typical centrosome of a mammalian somatic cell consists of a pair of centrioles and its associated pericentriolar material (PCM) (Figure 1).

Centrioles are cylindrical structures of approximately 500 nm in length and around 250 nm in diameter with a defined proximo-distal axis. Both centrioles are generally orthogonally oriented and interconnected at their proximal ends. Somatic centrioles are largely identical in morphology to the centrioles in male germ cells, consisting of nine microtubule triplets oriented in a pinwheel fashion [22]. In interphase, the centrosome functions as the main microtubule-organizing center of the cell, mainly by anchoring microtubule minus-ends via γ-tubulin ring complexes (γTuRCs) located in the PCM [23,24,25,26,27]. The major protein component of the γTuRC is γ-tubulin, which is predominantly localized in the PCM and functions in microtubule nucleation [28,29,30]. Since a structural organization in the PCM was hardly detectable for a long time, it was viewed as an amorphous cloud. However, the recent application of modern super-resolution microscopy techniques has changed this view. A highly ordered lattice structure within the PCM was first demonstrated by immunofluorescence image deconvolution. The lattice is formed by the centrosomal protein pericentrin (PCNT) that co-localized with γ-tubulin, the major protein component of γTuRCs [31,32]. Additionally, a higher-order structure of the PCM with a distinct protein composition adjacent to the centriole has been described [33,34]. Finally, the application of super-resolution microscopy revealed an organization of the PCM into domains with distinct molecular composition and architecture in both mammalian and *Drosophila* cells. In *Drosophila* cells, radial arrays extend outward from the proximal wall of the mother centriole. These radial fibrils are composed of pericentrin-like protein (PLP, Dplp) that most likely provides a scaffold for the organization of the outer PCM matrix [35]. Furthermore, PLP is exclusively associated with the mother centriole. In mammalian cells, distinct concentric domains populated by a specific set of proteins were identified around the proximal end of the mother centriole [36].

Centrioles duplicate synchronously with the cell cycle in that each preexisting/old centriole sprouts one new procentriole at its lateral side [37,38]. Due to the coordinated duplication cycle of the centrosome, which is under strict control of the cell cycle, each cell comprises one and only one centrosome in the G1-phase [39,40]. Duplication of centrioles starts in the G1 or S phase by the assembly of one procentriole oriented perpendicular along the long axis of each preexisting centriole. In the G2 phase, procentrioles elongate to reach their final size and each centriolar pair assembles a PCM. Finally, the two centrosomes separate, each comprising a pair of centrioles, an old or mother centriole and its newly generated daughter centriole, and its surrounding PCM. The two centrosomes eventually constitute the spindle poles to assemble the bipolar mitotic spindle and are subsequently distributed and transmitted into the two daughter cells. The centrosome duplication cycle ensures that each cell comprises two different centrioles: the younger or daughter centriole that was generated in the last cell cycle and the older or mother centriole originated at least one cell cycle earlier. Furthermore, the two centrioles differ not only in age but also in structure, protein composition, and function. The mother centriole comprises distal (DA) and subdistal (SDA) appendages at their distal end. SDAs function in nucleating and anchoring microtubules at their tip [41,42,43]. When the cell leaves the cell cycle to enter the G0 phase, the mother centriole is transformed into the basal body that assembles a ciliary axoneme at its distal end and anchors to the cell membrane, exposing the cilium into the cellular environment [44]. Assembly of ciliary microtubules starts at the distal end of the mother centriole/basal body, and it is here where the major protein component of the γTuRC, γ-tubulin, was detected within the core of the centriole [45]. Docking at the cell membrane takes place via the DAs, also named transition fibers in basal bodies, that pursue the ninefold symmetry of the triplet microtubules and have been described as radial protrusions resembling fingers or blades of a turbine [46,47]. DAs mediate the attachment of ciliary vesicles to the mother centriole and are, therefore, essential for ciliogenesis. SDAs consist of a conical striated stem attached to the two adjacent MT triplets of the centriole/basal body wall. SDAs of mother centrioles may coalesce to form the basal foot of the basal body as both appendages are mutually exclusive [48,49,50]. Furthermore, their number, thickness, and distribution along the proximo-distal axis of the mother centriole/basal body vary considerably. Up to nine SDAs could be present, whereas only one to two basal feet are found. Additionally, basal feet are larger than SDAs [51,52]. Basal feet and their regular position on the basal bodies of motile cilia, which are present in large numbers in specialized cells, define the polarization of the coordinated ciliary movement [53]. The formation of transition fibers and basal feet demands ODF2, which additionally mediates the orientation of basal feet [54,55]. Basal bodies and basal feet anchor MTs and that is consistent with the presence of γ-tubulin [56].

Furthermore, additional accessory structures associated with the basal bodies of multi-ciliated epithelial cells and the sensory connecting cilium of photoreceptor cells, the rootlets, have been described [56,57,58,59,60,61,62]. Rootlets are cytoskeleton-like structures of 80–100 nm in diameter extending from the proximal end of basal bodies toward the nucleus. They show regular cross-striation and are extensively cross-linked to intermediate filaments. In mammalian photoreceptor cells, rootlets associated with the basal body of the connecting sensory cilium are prominent structures. Their postulated function is to anchor and support cilia. A structural component of ciliary rootlets in murine photoreceptor cells and human T lymphoblastoid cells is rootletin [63,64]. Rootletin is distantly related to C-NAP1/CEP250 that functions in centriole cohesion by forming linker structures [41,65,66,67]. Likewise, rootletin (also named CROCC) is also involved in the formation of centriole-associated fibers and centrosome cohesion [68,69].

The centrosome is assembled by hundreds of proteins, among them α/β-tubulin dimers and γ-tubulin as the main proteins of centrioles and the PCM. The assembly of centrosomal proteins and the recruitment of the PCM around the centrioles is mediated by CEP192 and the centriolar satellite component PCM1, and centrosomal attachment of γ-tubulin is mediated by pericentrin and CDK5RAP2 [31,70,71,72,73,74,75,76]. Along with γ-tubulin, centrin (CETN, caltractin), belonging to the highly conserved EF-hand superfamily of Ca^2+^-binding proteins, is located in the centrosome and the distal lumen of centrioles and basal bodies [77,78,79,80]. CETN2, together with POC5, POC1B, and FAM161A, forms a scaffold that localizes along the inner wall of centrioles in their central and distal regions and keeps microtubule triplets together [81]. Furthermore, along with intrinsic centrosomal proteins, several proteins are only temporarily associated with the centrosome. It was, therefore, assumed that the centrosome may function as a hub in which signaling proteins and their relevant modifiers are locally concentrated to facilitate their interaction. The application of omics methods, especially proteomics, combined with the subsequent validation of the identified proteins by molecular and subcellular analyses enabled a comprehensive compilation of centrosomal proteins [64,82,83,84]. Centrosomal proteins were initially named as CEPx according to their molecular mass (xkDa). CEP proteins play vital roles in centriole biogenesis [85]. Furthermore, about 60% of centrosomal proteins have the propensity for coiled-coil domains and are, therefore, often named coiled-coil domain-containing of xkDa (CCDCx). The coiled-coil domain is, therefore, a common feature of centrosomal proteins, indicative of their structural role [82]. Centrosomal proteins often localize to specific substructures of the centrioles or basal bodies, thus indicating specialized functions and discriminating mother and daughter centrioles [86].

A distinguishing feature of the daughter centriole is the enrichment of centrobin (CNTROB), CEP120, and STIL [87,88,89,90]. Cilia formation is regulated by CP110 and CEP97 that are localized at the distal ends of centrioles [91,92]. Far more proteins have been identified as being enriched in the mother centriole/basal body and its appendages. CEP19, FOP (CEP43), CEP350, C2CD3, and chibby (CBY1) are enriched at the distal ends of the mother centriole [93,94,95]. The DAs of the mother centriole are characterized by the localization of C2CD3, CEP164, OFD1, CEP123 (CEP89/CCDC123), CEP83/CCDC41, SCLT1, and FBF1 [73,96,97,98,99]. Furthermore, a specific protein signature characterizes the SDAs comprising CEP170 [100], ODF2/cenexin [101,102,103], CEP128 [104], centriolin [105], ε-tubulin [106,107], and ninein [108,109,110,111]. The hierarchical assembly of DA– and SDA–proteins in the cell cycle has been figured out by super-resolution microscopy and revealed the central role of C2CD3 and ODF2/cenexin for DA and SDA assembly, respectively [46,47,112,113].

Centrosomal proteins can be functionally subdivided into different categories (Table 1): (1) those of the pericentriolar matrix that are mostly involved in microtubule anchorage and nucleation, and spindle formation; (2) proteins regulating centriole duplication, i.e., procentriole formation, elongation, and stabilization of centrioles; (3) centriole linker proteins that are involved in centriole cohesion and centrosome separation; and (4) proteins regulating assembly of cilia. An excellent overview of centrosomal protein interactions and their interconnection into functional complexes is found in [114]. Centriole duplication, and ciliogenesis and their related disorders (ciliopathies) are the topics of several excellent reviews, and we have to apologize for the impossibility to cite all of them. Instead, exemplary reviews are [40,115,116]. Considering the formation of the centriole-derived connecting piece, we will focus here on structural proteins intimately associated with the centrioles.

## 3. Building the Connecting Piece and Its Ultrastructure

**Figure 2:**
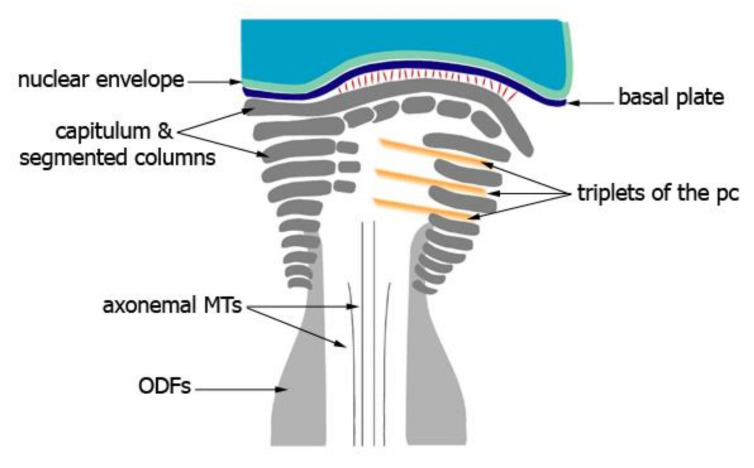
Schematic drawing of the connecting piece of a mature spermatozoon. The singlet MTs approaching the proximal centriole are visible. Striated/segmented columns fuse with the outer dense fibers. The articular structure of the capitulum is depicted. The convex capitulum lines the concave basal plate, a local thickening of the nuclear envelope, and is inserted into the nuclear implantation fossa. Thin fibers linking the capitulum and basal plate are shown in red. A typical distal centriole is no longer present. The proximal centriole disintegrates in rodent spermatozoa. MTs, microtubules; ODFs; outer dense fibers; pc, proximal centriole.

The connecting piece is a sophisticated structure that establishes the head-to-tail coupling apparatus (HTCA) and is, thus, situated in the neck region of the spermatozoon. The connecting piece is an articular structure formed by the convergence and fusion of nine longitudinal segmented columns. The segmented columns merge caudally with the nine outer dense fibers of the sperm tail and anteriorly with the capitulum, which is a dense articular structure positioned between the proximal centriole and the nucleus. The convex capitulum is inserted into a nuclear concavity forming the implantation fossa and is in close contact with the basal plate, a local thickening of the nuclear envelope. Thanks to many excellent ultrastructural studies of the connecting piece in spermatozoa of diverse animal species as well as its development during spermiogenesis, an overall and general picture can be figured out with some deviations due to species-specific modifications [117,118,119,120,121,122,123,124,125]. The depiction given here is mostly based on the development of the connecting piece in mouse and rat spermatozoa as originally described by Fawcett and Phillips [119] and Irons [125] (Figure 2).

**Figure 3:**
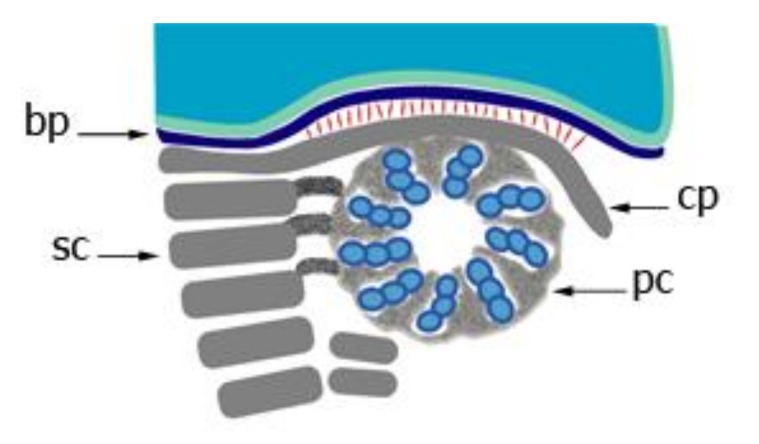
Schematic depiction of the developing connecting piece. Transverse section of the proximal centriole demonstrating the continuity of the dense material located in the space between the centriolar triplets with the future capitulum. This dense material fuses with the striated columns forming the capitulum. The capitulum is linked to the basal plate via thin fibers (in red). The basal plate is a local thickening of the nuclear envelope in the implantation fossa. bp basal plate, sc segmented columns, cp capitulum, pc proximal centriole.

The generation of the connecting piece as well as of the flagellum is initiated by the centrioles. The centrioles in germ cells and somatic cells are essentially identical in morphology consisting of nine triplet microtubules and are mostly present pairwise. A pair of centrioles, the diplosome, is also present in male germ cells. In mitotic and meiotic cells, centrosomes are located at the spindle poles and are involved in the formation of the spindle apparatus. Haploid spermatids harbor a pair of centrioles as well. The centriole pair is first located at the anterior region of the early spermatid in close vicinity to the Golgi apparatus and then migrates toward the posterior pole of the spermatid nucleus [119,125,126]. The axoneme is assembled from the distal centriole, which is oriented perpendicular to the cell surface [119]. At this early stage, outgrowth of the axoneme from the distal centriole, which has by now been transformed into the basal body, resembles that of primary cilia formation in somatic cells in which the basal body is also perpendicularly orientated toward the cell membrane. Axoneme formation from the distal centriole starts early in the cytodifferentiation of round spermatids into mature spermatozoa, that is, step 1 spermatids in the rat. In step 2 spermatids, the diplosome migrates toward the posterior nuclear region and reaches its final place by the beginning of the acrosome phase, that is, step 8 of spermiogenesis in the rat [125]. In contrast to somatic cells, the centrioles in spermatids also assemble a specialized structure, the connecting piece. The development of the connecting piece has been carefully analyzed by Fawcett and Phillips [119] in chinchilla, Chinese hamster, and mouse testes and by Irons [125] in the rat (Figure 3).

The first indication for its development is the accumulation of dense material immediately adjacent to the wall of both centrioles. At the proximal centriole, which is positioned juxta-nuclear and perpendicular to the long axis of the sperm flagellum, a sheet of dense material appears between the wall of the proximal centriole and the prospective implantation fossa of the nuclear membrane [119,125]. At this early stage, the generation of striated columns is already visible by rectangular densities located parallel to the wall of the distal centriole and extending toward the open end of the proximal centriole. Concomitantly, proximal and distal centrioles together with the attached flagellum relocate toward the nuclear membrane, eventually positioning the proximal centriole in the nuclear indentation. In this early stage, a local thickening of the cytoplasmic aspect of the nuclear envelope at the implantation fossa is already visible, forming the basal plate. The dense lamina surmounting the proximal centriole in spermatids and young spermatozoa is the anlage of the capitulum [119,125]. Throughout the acrosome phase, precisely throughout steps 8–12 of spermiogenesis in the rat, dense material progressively accumulates at the centriolar walls and gradually becomes organized into the capitulum and striated columns [125]. The dense lamina of the anlage of the capitulum is gradually transformed into the articular facet of the future capitulum by fusion with the dense material accumulating between the triplets of the proximal centriole and extending outward to reach the dense lamina [119,123]. Additionally, the dense material, which first accumulates between the triplets of the proximal centriole, also encroaches upon the lumen of the centriole, and the material from the posterior part of the proximal centriole coalesces with the developing striated columns [119]. Striated columns are assembled adjacent to the wall of the distal centriole on either side, originating from dense material that accumulates between the triplet microtubules of the distal centriole and extends outward in a radial fashion, forming the nine striated columns. Rod-like dense material is deposited between the axial pair of microtubules and the centriole wall [119,124]. Therefore, the cross-striated material that forms the bulk of the connecting piece is assembled from both centrioles.

Additionally, the anterior coalescence of material first assembled between specific triplet tubules of the distal centriole, forming the two thicker columns on opposite sides of the connecting piece, contributes to the formation of the final capitulum. These two columns are continuous anteriorly with the dense material from the juxta-nuclear centriole [119]. The five minor striated columns arise also from the contribution of both centrioles. The formation of the capitulum by the anterior coalescence of the striated columns has been described as a sharp bending of the segmented columns at different levels around the proximal centriole where they run in parallel to the long axis of the proximal centriole within the space of two adjacent microtubule triplets. The articular structure of the capitulum seems to be generated by the wrapping of segments of each column around the exterior of a corresponding triplet and penetrating the space between adjacent triplets of the proximal centriole [123]. In step 15 spermatids in the rat, the connecting piece is fully differentiated and the implantation fossa with the basal plate assumes its definitive shape, conforming to the contours of the capitulum [125]. Furthermore, the two central microtubules from the axoneme extend toward the proximal centriole and contact its wall. The articular surface of the capitulum of mature sperm is separated from the basal plate by a space of 400 Å traversed by fine filaments. These fine filaments are most likely the morphological equivalent of the head-to-tail linkage complex [7]. However, a direct contact of the capitulum and the basal plate at the periphery has also been described for some species [119]. The presence of the basal plate together with the connecting piece in decapitated monkey (*Macaca mulatta*) sperm flagella supports the notion that the basal plate is firmly attached to the connecting piece but not to the nuclear envelope [123].

The striated columns of the neck pass over into the ODFs of the sperm tail that accompanies the microtubule doublets of the axoneme. However, the striated columns are generated much earlier than the ODFs. The ODFs arise as thin filaments intimately associated with the tubule doublets of the axoneme along their entire length. As they become progressively thicker, they separate from the axonemal complex except at their terminal ends [119]. Finally, the ODFs fuse with the distal ends of the nine striated columns of the connecting piece [119].

As pointed out by Fawcett and Phillips [119], the mode of assembly of the striated columns of the connecting piece resembles that of satellite fibers and rootlets of epithelial cells that arise in close relation to centrioles. They have, therefore, suggested that the connecting piece of mammalian spermatozoa is homologous to centriolar satellites and rootlets of epithelial cells.

In the developing spermatids of a variety of mammalian species, the proximal centriole increases in length at its free distal end, forming a centriolar adjunct with a slightly different internal organization. In the late stages of spermiogenesis, the centriolar adjunct disappears and has not been found in mature spermatozoa of any other mammalian species besides humans [119,123,125]. Even in human spermatozoa, the centriolar adjunct is present only in part of them, whereas in patients with idiopathic male infertility, it is retained in almost all spermatozoa [127].

The persistence of proximal and distal centrioles in mature spermatozoa and sperm is heterogeneous between species. Thus, sperm with either both structurally recognizable centrioles, with only the proximal centriole, or even no typical centrioles have been described throughout the animal kingdom [128]. In the rat, the distal centriole is no longer identifiable by the time the spermatozoa reach the cauda epididymis [129]. The disintegration of the distal centriole during the testicular stage has also been observed in the mouse [130]. The distal centriole undergoes profound changes from its typical cylindrical form to an inverted cone and ultimately disappears. A distal centriole and thus a typical basal body at the base of the sperm tail is no longer present in mammalian sperm [119,124,131]. Since the cone-shaped region was only transiently observed, first in man and monkey spermatozoa by Zamboni and Stefanini [123], they suggested its renaming into transitional connecting centriole (TCC). In cross sections of the TCC, doublet and two singlet microtubules were identified and, depending on the plane of the cross-section, also triplet fibers, which are altogether reminiscent of centriolar and axonemal structures. Peripheral fibers and singlet fibers both extend toward the proximal centriole [123]. Thus, a distal centriole is not anymore present in its typical form in mature spermatozoa. Since the typical distal centriole is no longer present at the base of the flagellum, leaving only a vault and residual microtubules, it seems to be without any function. However, ultrastructural and molecular investigations revealed that the distal centriole in human and bovine sperm is not eliminated but instead profoundly remodeled, generating a novel atypical centriole that acts as a functional centriole in the zygote [132]. The atypical centriole is still attached to the base of the axoneme. It consists of doublet microtubules splayed outward, forming an inverted cone, flanked by rods [119,123,124,132].

The proximal centriole has been demonstrated in epididymal spermatozoa and sperm obtained from the seminal fluid of boar [121], bull [133], guinea pig [118], rabbit, monkeys, and man [123,134]. However, Ounjai et al. [135] could neither detect a distal nor a proximal centriole in bovine sperm. Woolley and Fawcett [129] detected the proximal centriole in late spermatids of rats but not in mature spermatozoa, indicating that it disappears before spermiation. The disappearance of the proximal centriole and its adjunct was later on narrowed down to the late step 17 spermatids in the rat [125]. Instead of a structurally recognizable centriole an empty space, the vault was left in the connecting piece. In the mouse, the proximal centriole degenerates in the epididymis [130]. Thus, in humans and most other mammals, the proximal centriole persists, whereas in rodent sperm both centrioles disintegrate [131].

## 4. The Protein Components of the Connecting Piece and Their Relevance for the Head-to-Tail Linkage and Male Fertility

A low percentage of morphologically abnormal spermatozoa are common in the semen of fertile men. However, a specific structural abnormality affecting all or most sperm in an infertile man is suspected of having a genetic origin, especially when detected in consanguineous sterile patients [136]. Although rarely found, a few types of such specific sperm defects have been described. Acephalic spermatozoa syndrome (ASS) is characterized by the separation of the sperm head from its tail. In several case reports of unrelated infertile men, it was shown that the ejaculate consists mainly of moving sperm tails, whereas heads are rarely found. In most cases, sperm decapitation occurs at the neck region. Ultrastructural studies have revealed that the loose tails comprise a regularly structured neck, including the proximal centriole, suggesting that the breakage point is anteriorly from the proximal centriole [137,138,139,140,141,142]. One patient was reported with sperm decapitation originating from the disconnection between the proximal and the distal centriole that, furthermore, was accompanied by an abnormal connecting piece [143]. Currently, a combination of morphological and molecular investigations successfully disclosed genes responsible for HTCA formation and its impairment in acephalic spermatozoa (Table 2). The generation of mice deficient for a specific gene is essential to finally prove that the gene/protein functions in HTCA formation and the subsequent identification of mutations in this specific gene in infertile men suffering from ASS support its relevance in humans. However, the current broad application of next-generation sequencing methods disclosed a whole range of mutations in many genes that have then been correlated with male infertility and sperm decapitation syndrome. Without demonstrating the effect of the loss of function or mutation of a respective gene in an animal experiment, all reported mutations are only correlations. It has therefore to be kept in mind that in this case, the detected mutations only indicate that the respective gene might be involved in HTCA formation.

As has been noted early, proteins that are incorporated into the developing neck region of steps 8 to 15 spermatids in the rat and afterward become permanently incorporated into this structure contain proline and cysteine [125]. Furthermore, it is well known that the proteins of the connecting piece, the ODFs, and the FS are insoluble and resistant against sodium dodecyl sulfate treatment and are stabilized by disulfide bonds generated by cysteine amino acids [191]. The treatment of rabbit spermatozoa with thiols caused head detachment, indicating the importance of disulfide bonds for the head–tail linkage [192]. Furthermore, cysteines can bind zinc ions that are enriched in the dense fiber-connecting piece fraction and appear to have a role in maintaining the sperm head–tail connection since tail detachment was observed after zinc extraction [193,194]. A major protein of the sperm tail ODFs with a high content of proline and cysteine capable of forming disulfide bonds is ODF1/HSPB10 [195,196,197]. The aforementioned findings pointed to ODF1 as an important HTCA component. Immune-EM confirmed the localization of ODF1 in the ODFs, segmented columns, the capitulum, and the basal plate of the HTCA that is also almost identical to the localization of ODF2 [144]. Targeted deletion of the *Odf1* gene in mice revealed infertility in males in the homozygous condition, whereas females were not affected at all [145]. ODF1-deficient spermatozoa are characterized by a disorganized tail and sperm decapitation, whereas heterozygous spermatozoa show a relaxation of the HTCA by an enlarged distance between the capitulum and the basal plate [146]. A reduction in ODF1 protein was found in the semen of infertile men, and after stress treatment, sperm easily decapitate [147]. All in all, ODF1 is essential for the tight linkage of the sperm head to the tail. Another predominant protein of the ODFs is ODF2, which localizes to almost the same substructures in spermatozoa as ODF1. ODF2 is a structural protein consisting essentially of coiled-coil domains [149,150]. Furthermore, ODF2 has been identified as a centrosomal scaffold protein that is preferentially associated with the mother centriole and was, hence, named cenexin [101,102]. When absent, distal and subdistal appendages on mother centrioles are missing and primary cilia cannot be formed [103]. Targeted deletion of *Odf2* in mice caused embryonic lethality, but chimeric male mice made of a gene trap insertion into the *Odf2* gene are infertile, and male mice with a heterozygous deletion of *Odf2* are infertile caused by neck–midpiece separation in sperm [151,152,153]. ODF1, as well as ODF2, interacts with CCDC42, a coiled-coil domain-containing protein that localizes to the manchette, connecting piece, and tail, which is essential for male fertility [148]. Targeted deletion of CCDC42 in mice caused male sterility with malformed spermatid heads, dislocation of the HTCA from the implantation fossa, multiplicity of HTCAs, and missing axonemes [158]. Although neither ODF1 nor CCDC42 were identified in the proteomics screens of centrosomal proteins [64,82], we found colocalization of CCDC42 together with ODF1 and ODF2 at the centrosome [148]. Since all three are cytoskeletal proteins, the quest for binding partners that link the cytoskeletal structure to the nuclear envelope is mandatory. In somatic cells, the LINC complex, comprising inner nuclear membrane proteins of the SUN-domain family and outer nuclear membrane proteins of the KASH-domain family, bridges the nuclear envelope and ***li***nks the ***n***ucleoskeleton to the ***c***ytoskeleton [198]. Besides the two ubiquitously expressed SUN-domain proteins, SUN1 and SUN2, three testis-specific proteins exist: SUN3, SUN4/SPAG4, and SUN5/SPAG4L [199,200]. SUN4/SPAG4 is a described binding partner of ODF1 and was, therefore, expected to be the missing link of the head-to-tail coupling [159]. However, although *Spag4*-deficient male mice are infertile with severe defects in sperm head formation, only mild effects on the linkage between head and tail were found [160,161,162]. In contrast, disruption of *Sun5/Spag4l* in mice caused male infertility by sperm decapitation [163]. Additionally, several mutations in *SUN5* have been identified in infertile men suffering from ASS [164,165,166]. As SUN-domain proteins are supposed to be inner nuclear membrane proteins with their N-terminal ends exposed toward the nucleoskeleton and their C-terminal ends situated inside the perinuclear space interacting with the C-terminal ends of the outer nuclear membrane proteins of the KASH-domain family, direct interaction with cytoskeletal proteins is hardly imaginable. Thus, a careful reinvestigation of the topological orientation of SUN-domain proteins within the nuclear envelope is mandatory. Bridging the nuclear envelope to link the nucleoskeleton to the cytoskeleton is achieved by the binding to KASH-domain proteins that interact with their N-terminal ends with the cytoskeleton. Although currently no specific KASH-domain proteins have been identified that are involved in HTCA formation, SUN5 interacts with the KASH-domain protein nesprin3 in spermatozoa, indicating that the HTCA comprises at least the SUN5/nesprin3 LINC complex [167,201,202].

**Figure 4:**
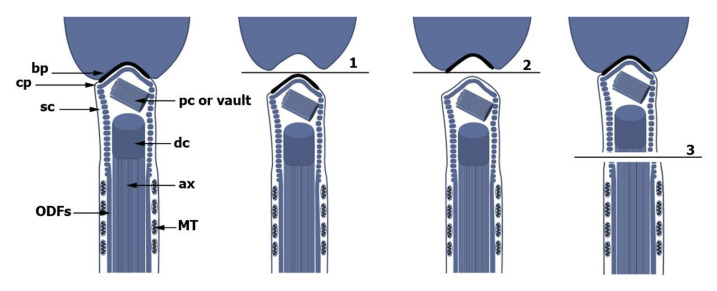
Sperm decapitation occurs at three different positions. Compared to the intact spermatozoon (left), breakage line 1 is between the nuclear envelope and the basal plate and has been observed in *Odf3*/*Pmfbp1*-ko mice. The basal plate thus remains attached to the tail. Breakage line 2 separates the basal plate from the capitulum. The basal plate remains therefore associated with the nucleus. This is the most common case. Breakage line 3 separates the neck region, which remains attached to the nucleus, from the mid-piece, and was found in spermatozoa with a heterozygous deletion of *Odf2*. Since the scheme was assembled from data obtained from infertile patients with ASS as well as from mice, the positions of distal and proximal centrioles, dc and pc, respectively, are shown. However, it has to be mentioned that typical distal centrioles are no longer present and that in mice the proximal centriole disintegrates, too. Instead, atypical distal centrioles and a vault in the place of the former proximal centriole (pc) in mice are found. ODFs, outer dense fibers; MT, microtubules; ax, axoneme; sc, segmented columns; cp, capitulum; bp, basal plate.

The localization of the outer dense fiber proteins ODF1 and ODF2 to striated columns, the capitulum, and the basal plate indicates that neck structures and ODFs share a similar protein composition. ODF3 is another ODF protein that is involved in HTCA formation [154]. Rat ODF3 (AAC72233.2) is identical to rat PMFBP1 (XP006255636.3) besides the N-terminal part of ~60 amino acids that are missing in ODF3 and is 70% identical to human PMFBP1 (NP_112583.2). Crispr/Cas-mediated deletion of *Odf3*/*Pmfbp1* in mice caused acephalic spermatozoa [155]. Since the basal plate remains attached to the capitulum and not the nuclear envelope, the breakage line is in between, indicating that ODF3 is involved in the linkage of the basal plate to the nuclear envelope [155]. Mutations in *ODF3*/*PMFBP1* have also been reported in consanguineous families with ASS [155,156,157]. ODF3/PMFBP1 is also known as polyamine modulated factor 1 binding protein 1, and polyamines seem to be critically involved in sperm head–tail linkage. The intracellular concentration of polyamines is controlled by ornithine decarboxylase antizyme OAZ-t/OAZ3 that binds and inactivates ornithine decarboxylase. OAZ-t/OAZ3 is specifically expressed in spermatids. Homozygous *Oaz-t*-mutant male mice are infertile due to easy sperm decapitation, indicating that OAZ-t/OAZ3 is essential for the formation of a rigid head-tail junction. The breakage line in OAZ-t/OAZ3 deficient sperm is between the basal plate and the capitulum [168] (Figure 4).

Speriolin/SPATC1 and speriolin-like protein/SPATC1L are both located in the neck region. Speriolin is concentrated at the centrosome of spermatocytes and spermatids and surrounds the intact proximal centriole in human sperm and the periphery of the disordered distal centriole in mouse sperm [169,170]. Neither has the involvement of speriolin in HTCA formation been investigated, nor have speriolin mutations been reported in infertile men with ASS, whereas a Crispr/Cas-mediated knockout of *Spatc1l* caused sperm decapitation and male sterility. Since the basal plate remains associated with the tailless head, the breakage line lies between the basal plate and the capitulum [171]. Whole-exome sequencing revealed biallelic mutations in *SPATC1L* in infertile men with ASS [172]. SPATA6 resides exclusively in the connecting piece and localizes to striated columns and capitulum. Disruption of *Spata6* in mice caused severe disturbance of connecting piece and tail structures. Although the PC was present in spermatids, segmented columns, capitulum, and mitochondria are missing. Thus, male mice are sterile with decapitated sperm [173]. Sperm decapitation or easily decapitation in mice has also been reported for the functional inactivation of PRSS21 (glycosylphosphatidylinositol-anchored serine protease, or testisin) and SPAG6 [174,175]. SPAG6 is a component of the central apparatus of 9 + 2 axonemes, and *Spag6*-deficient mice show disturbed spermatogenesis with abnormal germ cell morphology and disorganized flagellar structures [176].

The manchette is essential for the reshaping of the spermatid nucleus, and its microtubules function as a track for the storage and delivery of molecules destined to the formation of the flagellum and possibly also the connecting piece [9,12]. Impairment of the manchette and the IMT thus affects the correct formation of the sperm tail and could also be detrimental to HTCA formation [203]. Manchette formation is compromised in the *azh* (abnormal spermatozoon head shape) mouse caused by a truncating mutation in HOOK1 [177]. The HOOK family of proteins is involved in endosomal trafficking and functions as adaptors and activators for the minus-end-directed motor protein dynein [204]. HOOK2 localizes to the centrosome and interacts with centriolin/CEP110 in somatic cells [205]. We have shown that HOOK1 is also a centrosomal protein in NIH3T3 cells by using an antibody directed against the HOOK1-specific C-terminal end. Furthermore, pull-down experiments using HOOK1-MBP and ODF2-GST fusion proteins have demonstrated a direct interaction between HOOK1 and ODF2 (S. Hoyer-Fender and J. Neesen, unpublished). The truncated HOOK1 in the *azh* mouse provoked easy sperm decapitation, indicating a compromised sperm head–tail connection [177]. Furthermore, whole-genome sequencing of patients with ASS revealed mutations in *HOOK1* [178]. HOOK1 interacts with RIM-BP3 that is essential for correct manchette formation and the rigid head-to-tail linkage, as observed by the targeted deletion in mice [179]. Genetic inactivation of FAM46C in mice, which is specifically located to the manchette, caused the incomplete formation of segmented columns and capitulum, and sperm decapitation [180]. Centrobin/CNTROB, which is involved in centriole duplication and stability (Table 1) localizes to the manchette, capitulum, DC, and PC. Truncation of the protein by a retroviral insertion into intron 10 of the gene in the *hd* rat caused sperm decapitation. However, a full-length cDNA was not able to rescue the sperm phenotype. It is, therefore, not clear whether inactivation of CNTROB is causative for sperm decapitation and, thus, if it is essential for the sperm head–tail linkage complex [89,181,182].

A Crispr/Cas-mediated depletion of TSGA10, a mitochondrial-associated protein, causes infertility in heterozygous male mice, with reduced sperm count and disordered mitochondrial sheath formation. Infertile patients suffering from ASS have revealed mutations in *TSGA10* by whole-exome sequencing [156,157,183,184]. TSGA10 has been identified as a centrosomal and basal body protein that interacts with ODF2 [185]. Sperm decapitation has also been observed by conditional inactivation of the serine-threonine kinase fused, which interacts with ODF1 and KIF27 [186], and by *Arl3*-siRNA injection into the testis [187]. Mutations in patients with ASS have been detected in BRDT, which functions in transcriptional repression during spermatogenesis, and in CEP112 [188,189,190].

As already outlined, one or both typical centrioles in sperm disappear, which is known as centrosome reduction. During reduction, the typical centriole structure disintegrates and associated proteins are eliminated. In the expected place of the centriole, an empty space, the vault, appears. However, microtubular structures and rod-like dense material, deposited between the axial pair of microtubules and the centriole wall, have been observed in the vault previously occupied by the distal centriole [119,123,124,132]. Ultrastructural and molecular investigations demonstrated that the distal centriole is morphologically remodeled into an atypical distal centriole with splayed doublet microtubules and rods at the base of the axoneme comprising centriole luminal proteins [132]. Additionally, centrosome reduction includes also the loss of pericentriolar material and the elimination of centrosomal proteins as well as the attenuation of microtubule nucleation. In elongating spermatids of the mouse both, the proximal and distal centriole harbor centrin (CETN). However, neither γ-tubulin nor centrin could be detected in mature spermatozoa, in which both centrioles are degenerated, whereas in the mature spermatozoa of rhesus monkeys, in which the proximal centriole remains intact, γ-tubulin is lost but not centrin [206,207]. A refined analysis detected both, CETN1 and the centriole marker CETN3, in the proximal and distal centrioles in round spermatids of the mouse as well as in the centriolar adjunct. CETN1 depletion caused male infertility with abnormally shaped sperm heads, and a reduction or even absence of sperm tails, disorganization of centriole arrangement, and failures in the basal body–nucleus connection [208].

Centriole reduction is not at all associated with the elimination of centrosomal proteins. Proteomic analysis of the isolated bovine sperm centrosome identified 364 proteins by mass spectrometry. Comparative bioinformatics taking into consideration previous proteomics and genomics studies annotated 60% of them as already identified centrosomal proteins. Additionally, novel previously uncharacterized centrosomal components could be identified by their subcellular localization in cycling cells [209]. Bovine sperm centrioles, comprising only the typical proximal centriole, were isolated by sonication-mediated decapitation, isolation of the tail fraction, and enrichment for centrioles by sequential protein extraction. Since decapitation was induced by sonication, the breakage line is not known. It has, therefore, to be considered that essential structures and proteins of the HTCA eventually remain associated with the nucleus and thus might be missing in the proteome.

A comprehensive cytological investigation analyzing the presence and distribution of centrosomal proteins in human and bovine sperm revealed that the sperm centrosomes differ in protein composition from that of a typical somatic centrosome. Only a subset of centrosomal proteins could be identified. Furthermore, the proximal (PC) centriole and the degenerated distal centriole (DC) differ in protein composition and concentration [132]. Some typical centrosomal proteins were even not found in the neck region and the proximal centriole of human sperm, including the PCM components γ-tubulin, PCNT, and PCM-1, proteins involved in centriole duplication as CENTR3, STIL, SPICE1, SAS6, CEP152, CEP192, CEP295, and POC1A, or the distal appendage proteins CEP89/CCDC123 and FBF1 [132], albeit POC1A was found in the bovine sperm centriole proteome [209]. However, in contrast to previous observations, CETN1/2 was found to equally label both, the PC and the degenerated DC, in ejaculated human sperm. The DC, furthermore, showed enrichment of POC1B, which has also been detected by Firat-Karalar et al. [209] in the bovine sperm centriole proteome, and the location of the transition zone protein CEP290 at the junction between the DC and the axoneme. The presence of CEP164 and CDK5RAP2 in the striated columns and the capitulum indicates their close association with the centriolar microtubules and suggests that the neck region has emanated from the PCM [132]. CEP126, CEP120, and Ana/RTTN/rotatin, which are all related to MT, centriole assembly, and ciliogenesis, are located to the capitulum, as well as CNTROB, CDK5RAP2/CEP215, and SFI1 [132]. POC5, CEP63, CEP76, CEP135, and OFD1 are found in both centrioles, mostly with a preference for the PC, and CPAP additionally in the PCM. Of the centrosomal core module consisting of CEP192/CEP152/CEP63/CPAP, required for centriole duplication, the two essential proteins CEP192 and CEP152 are missing in the neck region [132]. Several centrosomal proteins investigated have even not been found by immune-cytology. However, albeit problems with antibody accessibility and quality might be considered for the detection failure, some of the proteins might also be of low abundance and thus below the detection limit. In this regard, it has to be mentioned that only POC1B could be detected in the bovine sperm centriole proteome by Firat-Karalar et al. [209].

## 5. Conclusions

The HTCA develops as a specialized and highly modified derivative of the centrosome. Molecular, cytological, and genetic investigations in experimental animals as well as next-generation sequencing efforts have revealed several genes that are essential for rigid HTCA formation. The majority of them seem to have an exclusive function in the HTCA and have not been detected in the centrosomal proteome. However, the centrosome of somatic cells, as well as of the sperm centrioles, comprise hundreds of proteins, mostly uncharacterized. Since the centrosome is a non-membraneous organelle, the unambiguous identification of intrinsic proteins by proteomics is challenging. The isolation procedure of centrosomes or sperm centrioles is prone not only to contamination with cellular proteins but also to the loss of essential centrosomal proteins. That might be exemplified by the reported list of sperm centriole proteins that comprise 364 proteins but miss most of the centrosomal proteins mentioned in Table 1. Furthermore, albeit cytological investigations by Fishman et al. revealed several centrosomal proteins in the sperm linkage complex, they were not detected in the bovine sperm centriole proteome besides POC1B. To uncover the complete protein constitution of the HTCA demands the combination of molecular, biochemical, and immunological methods, together with the disclosure of mutations by whole-exome/genome sequencing of infertile men with ASS. Furthermore, implementation of testicular organoids and in vitro generated spermatozoa combined with Crisp/Cas- or RNAi-mediated depletion might help identifying novel HTCA components, reducing also the consumption of laboratory animals. Additionally, since ODFs and HTCA seem to share their protein composition, further ODF proteins should be considered as essential HTCA components.

## Figures and Tables

**Table 1:** Localization and function of characterized centrosomal proteins. Proteins relevant for cilia formation are largely excluded.

Category	Protein	Function
**PCM Formation**	SAS4/CPAP	Centriole assembly
	CEP135	Centriole assembly
	CEP295	PCM assembly
	CEP152/asterless/Asl	PLK4 recruitment
	CEP192	PLK4 recruitment, PCM assembly
	NEDD1	Adapter of CEP192, binds to γ-TuRCs
	CDK5RAP2/CEP215	PCM, binds to γ-TuRCs
	PCNT/pericentrin	PCM, binds to γ-TuRCs
	CEP63	Binds CEP152 and CEP57
	CEP57	Anchors CEP63/CEP152 complex to the centriole
	PCM-1	Centriolar satellites
**Centriole Lumen**	POC1B	
**Scaffold**	POC5	
	CETN/centrin	
	FAM161A	
	WDR90	
**Centriole Duplication**	CEP192	PLK4 recruitment
	CEP152	PLK4 recruitment, centriole length control
	PLK4	
	STIL	Procentriole formation, cartwheel
	SAS6	Procentriole formation, cartwheel
	CEP135	Procentriole formation
	CPAP/CENPJ/SAS4	Procentriole formation, centriole length control
		Procentriole elongation
	CNTROB/centrobin	Centriole length control
	CEP120/CCDC100	Centriole length control
	SPICE1/CCDC52	Centriole length control
	POC1A, POC1B	Centriole length control
	POC5/C50rf37	Centriole length control
	CEP295	Centriole length control
	CP110	Centriole length control
	CEP97	Regulation of centriole duplication
	CEP76	Centriole duplication
	CEP72	
	CEP63	
	CEP57/translokin	
	CCDC57	
	WDR62	Centriole duplication, centriolar satellites
	CEP131/AZI1	Centriole length
	RTTN, PPP1R35, C2CD3	Centriole stability
	ε-Tubulin, ό-tubuline, TEDC1, TEDC2	
	RTTN/rotatin	
**Linker at the Proximal Ends of MC and DC**	Rootletin	
	C-NAP1/CEP250	
	CNTLN/centlein	
	CEP68	
	CG-NAP	
	LRRC45	
	β-Catenin	
**Distal Centriole Ends**	OFD1	Centriole stabilization
	CP110	
	CEP97	
	CEP78	
	CETN	
	CEP19	Distal ends of mother centriole
	CEP350	Distal ends of mother centriole
	C2CD3	Distal ends of mother centriole
	CEP43, CBY1/Chibby	Distal ends of mother centriole, centriolar satellites
**Daughter Centriole**	CNTROB	
	CEP120	
	STIL	
**Distal Appendages**	CEP164	Ciliogenesis
**of Mother Centriole**	CEP89/CEP123/CCDC123	
	CEP83/CCDC41	
	FBF1	
	SCLT1	
	C2CD3	
	OFD1	
**Subdistal Appendages of Mother Centriole**	ODF2/cenexin	MT anchoring
	TCHP/trichoplein	
	NIN/ninein	
	CEP170/FAM68A	
	CEP128	
	CEP110/Centriolin/CEP1/CNTRL	
	CCDC120	
	CCDC68	
	CCDC61/hVFL3	
	Nde1	
	ε-Tubulin/TUBE1	
	CC2D2A	

PCM, pericentriolar material; MC, mother centriole; DC, daughter centriole.

**Table 2:** Proteins essential for the tight linkage of sperm head and tail.

Protein	Centrosomal Localization and Function	Localization in Spermatozoa	Phenotype of Knockout Animals	Mutations in Humans	References
ODF1	Centrosome in NIH3T3 cells.	Manchette, HTCA, and flagellum/ODFs. ODFs, segmented columns, capitulum, and basal plate by IEM. Interacts with ODF2, CCDC42.	*Odf1* ko by HR. Male mice are infertile with disorganized sperm tails and sperm decapitation. Weakened head to tail linkage in the heterozygous condition.	Easy sperm decapitation in infertile men with a reduction in ODF1 protein.	[144,145,146,147,148]
ODF2	PCM, mother centriole, SDAs. Essential for primary cilia formation; centriole cohesion. Interacts with ODF1, CCDC42, CEP128, ß-catenin, trichoplein.	Flagellum/ODFs. ODFs, segmented columns, capitulum, and basal plate by IEM.	Chimaeric male mice of high percentage chimaerism, generated of *Odf2* XL169 ES cells, in which the ß-neo gene-trap cassette is inserted into the *Odf2* gene, are infertile. Infertility due to bent tails, missing or thinning of individual ODFs, absence of one or more axonemal MT doublets.		[101,102,103,112,149,150,151,152,153]
ODF3/PMFBP1		Flagellum/ODFs, HTCA. Cooperates with SUN5 and SPATA6.	Crispr/Cas-mediated ko. Male mice are infertile. Acephalic spermatozoa. Basal plate attached to the tail, not the nucleus.	Mutations reported in consanguineous families with ASS.	[154,155,156,157]
CCDC42	Centrosome and the basal body in somatic cells.	Manchette, connecting piece and tail. Interacts with ODF1 and ODF2.	*Ccdc42* ko mice by HR (null allele). Male sterility. Elongated spermatids with abnormally shaped heads, dislocation of the HTCA from the implantation fossa, and multiplicity of HTCAs. No MT axonemes observed from the HTCA.		[148,158]
SUN4/SPAG4	SUN-domain-containing protein, testis-specific, nuclear envelope.		Male infertility in *Sun4*-deficient mice. Defects in sperm head formation. Required for tight head-to-tail linkage in sperm, but HTCA is still present.		[159,160,161,162]
SUN5/ SPAG4L/4L-2	SUN-domain-containing protein, testis-specific, nuclear envelope.	Interacts with nesprin3.	TALEN-generated *Sun5* ko mice by targeting exon 4). Male infertility with decapitated spermatozoa. Basal plate associated with the capitulum.	Approximately 33-47% of patients with ASS have mutations in *SUN5*.	[163,164,165,166,167]
OAZ-t/OAZ3		Specifically expressed in spermatids.	Homozygous mutant males are infertile due to easy sperm decapitation. Basal plate remains associated with the nucleus.		[168]
Speriolin (*Sprn, Spatc1, Spata15*)		Concentrated at the centrosome of spermatocytes and spermatids. Localized in the neck region: surrounds the intact proximal centriole in human sperm, and the periphery of the dis-ordered distal centriole in mouse sperm.	Not reported	Not reported	[169,170]
Speriolin-like protein (*Spatc1l*, C21orf56)		Expression starts in the round spermatid stage in mice. Localized at the neck region in testicular sperm of mice. Associates with the regulatory subunit of cAMP-dependent protein kinase A (PKA). Interacts with capping protein muscle Z-line beta (CAPZB).	Crispr/Cas-mediated ko mice. Male sterility. Sperm decapitation. Separation between the basal plate and the capitulum.		[171,172]
SPATA6/SRF1/HASH		Striated columns, capitulum	Ko mice are male sterile. Acephalic spermatozoa, impaired development of the connecting piece (by TEM). pc present but segmented columns, capitulum and mitochondrial sheath are lacking. Disturbance of the tail structure (ODFs, MT).		[173]
PRSS21 /testisin			Targeted disruption of the coding sequence by HR. PRSS21 deficient spermatozoa show decreased motility, angulated and curled tails, fragile necks and easily decapitated spermatozoa.		[174]
SPAG6/PF16/CFAP194		Component of the central apparatus of the 9+2 axoneme.	*Spag6* ko by HR. *Spag6*-deficient testes showed abnormal spermatogenesis with abnormalities in male germ cell morphology. Disorganized flagellar structures and frequent loss of the sperm head. *Spag6*-deficient mice have further cilia-related disorders (i.a. hydrocephalus).		[175,176]
HOOK1	Centrosome in NIH3T3 cells.	Predominantly expressed in haploid male germ cells. Interacts with CCDC81, and ODF2.	HOOK1 mutation causative for the *azh* phenotype in mice, with head and tail abnormalities, and sperm decapitation.	Whole genome sequencing of patients with decapitated and decaudated spermatozoa revealed mutations in *HOOK1*.	[177,178]
RIM-BP3	RIM-BPs as adaptors in the process of vesicle fusion and release.	Manchette associated, interacts with HOOK1. Expressed almost exclusively in the testis.	Targeted deletion in mice revealed abnormal sperm heads and tail detachment.		[179]
FAM46C		Specifically located to the manchette of mouse spermatids.	Crispr/Cas-mediated ko mice. Male sterility with headless spermatozoa. In step13-16 spermatids, abnormal connecting pieces with incomplete segmented columns and capitulum.		[180]
Centrobin/ CNTROB/NIP2	Centriole duplication and stability	Capitulum, dc and pc. Localizes to the manchette, centrosome, and marginal ring of the acroplaxome.	Truncated CNTROB protein by insertion of a retrovirus in intron 10 of the gene in the *hd* rat (hypodactylous). Disruption of the HTCA and sperm decapitation. A full- length cDNA in testis did rescue limb malformation but not decapitation. CNTROB might be required for the stabilisation of the attachment of the sperm head to the flagellum.		[89,181,182]
TSGA10 (CEP4L, Cancer/testis antigen 79, CT79, SPGF26)			Crispr/Cas-mediated ko mice. Heterozygous male mice are infertile with reduced sperm count and disordered mitochondrial sheath formation.	Whole exome sequencing revealed mutations in ASS patients.	[157,183,184,185]
Fu/fused	Serine-threonine kinase	Interacts with ODF1, and KIF27.	Conditional inactivation caused sperm decapitation, i.a.		[186]
ADP-ribosylation factor-like 3 (ARL3)	Ras-related small GTP-binding protein.	Manchette.	siRNA injection into testis provoked abnormal head shape, lasso-like coiled tails, and sperm decapitation.		[187]
BRDT (cancer/testis antigen 9, RING3-like protein, BRD6, SPGF21)	BRDT (Bromo-domain testis associated) involved in gene repression.	BRDT expression restricted to male germ cells, specifically to pachytene/diplotene spermatocytes and early spermatids.	Loss of the first bromodomain of BRDT by targeted mutagenesis resulted in sterility and abnormalities in spermiogenesis.	Mutations in male patients with ASS.	[188,189]
CEP112/ CCDC46			Not reported.	Mutations found in male patients with ASS by whole exome sequencing.	[190]

ko, knockout; HR, homologous recombination; MT, microtubule; ODF, outer dense fiber; PCM, pericentriolar material; pc, proximal centriole; dc, distal centriole; DA, distal appendage; SDA, subdistal appendage; IEM, immune-electron microscopy; TEM, transmission electron microscopy.
